# Toxicity of fluralaner, a companion animal insecticide, relative to industry-leading agricultural insecticides against resistant and susceptible strains of filth flies

**DOI:** 10.1038/s41598-020-68121-z

**Published:** 2020-07-07

**Authors:** Edwin R. Burgess, Christopher J. Geden, Kimberly H. Lohmeyer, B. H. King, Erika T. Machtinger, Jeffrey G. Scott

**Affiliations:** 1 Department of Biological Sciences, Northern Illinois University, DeKalb, IL 60115 USA; 2 Center for Medical, Agricultural, and Veterinary Entomology, USDA-ARS, Gainesville, FL 32608 USA; 3 Knipling-Bushland U.S. Livestock Insects Research Laboratory, USDA-ARS, Kerrville, TX 78028 USA; 4 Department of Entomology, Pennsylvania State University, University Park, PA 16802 USA; 5 Department of Entomology, Cornell University, Ithaca, NY 14853 USA

**Keywords:** Entomology, Toxicology

## Abstract

Filth flies cause billions of dollars of losses annually to the animal production industry. Fluralaner is a relatively new pesticide currently sold for control of fleas, ticks, and mites on companion animals and poultry. We examined the efficacy of fluralaner against three species of filth flies. Insecticide-susceptible horn flies and stable flies were tested topically. Fluralaner outperformed permethrin by > 2-fold for the horn flies but underperformed permethrin by > 45-fold for stable flies at 24 h. House flies were tested topically with fluralaner in comparison to permethrin at 48 h and orally with fluralaner in comparison to imidacloprid at 24 h. Topical fluralaner was 6- to 28-fold as toxic as permethrin in four pyrethroid-resistant strains and not significantly less toxic than permethrin in a susceptible strain and a mildly pyrethroid-resistant strain. There was slight cross-resistance between topically applied fluralaner and permethrin in all five insecticide-resistant strains tested. Oral fluralaner was more toxic than imidacloprid in all four house fly strains tested, 9- to 118-fold as toxic. Oral cross-resistance between imidacloprid and fluralaner was not detected, but imidacloprid resistance was not high in any of the tested strains. Fluralaner shows promise for control of horn flies and house flies.

## Introduction

Filth flies (Diptera: Muscidae) are perhaps the greatest arthropod pest of animal production worldwide, causing billions of dollars (USD) in economic losses each year [e.g.^1–3^]. Animal production facilities can provide ideal conditions for filth fly development, promoting rapid population increases. Among the most important filth flies in the United States are the horn fly, *Haematobia irritans*, the stable fly, *Stomoxys calcitrans*, and the house fly, *Musca domestica*. Horn flies and stable flies are obligate blood feeders that deliver a painful bite, which contributes to decreases in cattle weight gain^2,4–6^ and milk production^2,7–9^. The house fly is a vector of many microorganisms of veterinary concern, including those that cause habronemiasis in equines^10^, mastitis in cattle^11^, and necrotic enteritis in poultry^12^. In addition, antibiotic-resistant bacteria can be carried by house flies^13–17^.

Chemical control historically has been employed for quick reduction of filth fly populations. Pyrethroids and their natural counterpart, pyrethrins, have been widely used for decades and are still widely used as sprays, dusts, and pour-ons (topical applications of insecticide applied to an animal’s coat or skin)^18^. As a result, strong selection for pyrethroid resistance has led to some populations of filth flies with no detectable susceptible alleles remaining^19^. For house flies, sugar-based toxic baits also have been used, including methomyl (carbamate), imidacloprid, and nithiazine (both neonicotinoids), but resistance has evolved to these insecticides as well^20–24^. In addition, effects on non-target beneficial organisms, such as pollinators, and perception of those effects is a problem. The hope is that new control chemistries may address these issues and that the alternation of insecticides can help stall the evolution of resistance^25^. Within the past decade, cyantraniliprole bait (Zyrox Fly) is the only new insecticide that has a mode of action different from previously existing filth fly insecticides^26^.

Fluralaner is a relatively new insecticide and acaricide belonging to the isoxazoline class (IRAC group 30). It was first registered with the United States Food and Drug Administration (FDA) in 2014 (NADA 141–426). The isoxazolines act as antagonists to the glutamate- and γ-aminobutyric acid-gated (GABA) chloride channels^27^, a target site that is different from pyrethroids, carbamates, and neonicotinoids. Fluralaner is currently used in the medication Bravecto, which is available as a topical or as a chewable tablet for flea and tick control in companion animals^28–30^. It is also formulated in Exzolt, an orally administered systemic for control of red mite (*Dermanyssus gallinae*) and northern fowl mite (*Ornithonyssus sylviarum*) in poultry^31,32^. Fluralaner also is effective against some other arthropods of medical and veterinary concern, e.g., certain mosquito larvae, sheep blow fly larvae and certain ticks of animal production concern^27,33^. Oral treatments of fluralaner even have been proposed to control vector-borne diseases in humans^34^. To date there are no published efficacy comparisons of fluralaner to currently-used topical and oral active ingredients against adult filth flies. Against adult stable flies fed spiked blood meals, fluralaner shows similar effectiveness as fipronil, but less than the pyrethroid deltamethrin^27^. Neither fipronil nor deltamethrin are currently used in products designed to end up in the blood meal of biting flies, such as stable flies or horn flies.

Permethrin, a pyrethroid, and imidacloprid, a neonicotinoid, are two common active ingredients for filth fly control. In this study, we compared the topical toxicity of fluralaner to that of permethrin in horn flies, in stable flies, and in six house fly strains with varying degrees of permethrin and/or imidacloprid resistance (Table 1). The comparison to permethrin was based on topical application because permethrin is not used in baits but in space sprays and pour-ons. We also compared the oral toxicity of fluralaner to that of imidacloprid in four strains of house flies with varying degrees of permethrin and/or imidacloprid resistance. Oral exposure was used to assess potential for use in a house fly bait.

**Table 1:** Species and strains of filth flies tested with fluralaner.

Species	Strain	Origin	Resistance profile	Dry weight of flies (mean ± SE in mg/fly)
Horn fly	USDA, Kerrville	Collected from Lake Jackson, TX in 1968	Insecticide-susceptible^55–57^	
Stable fly	USDA, CMAVE	Maintained at Gainesville, FL. Established in 1970’s	Insecticide-susceptible^42^	
House fly	NIU	Maintained at Northern Illinois University for > 25 years	Insecticide-susceptible, based on sequencing (Results) and lack of exposure to pesticides	5.04 ± 0.11
	KS17	Collected from Riley Co., KS in June 2017	Pyrethroid-resistant with mutations in the cytochrome P450 *CYP6D1v1* gene, several acetylcholinesterase mutations known to confer pesticide resistance, and several *kdr* mutations^19^	1.62 ± 0.07
	USDA-R	Maintained at Gainesville, FL for > 30 years	Pyrethroid-resistant with unknown mechanisms	3.77 ± 0.11
	USDA-mixed	Collected from Beatrice, NE, San Jacinto, CA, Bell FL, Morris, and Kirkoven, MN in 2014 and 2015. Mixed in 2017. Maintained at USDA-ARS-CMAVE	Pyrethroid-resistant but unknown mechanisms. Unknown insecticide exposure history	4.15 ± 0.10
	PA-mixed	Mixture of five populations collected from geographically separated animal facilities in Centre, Lancaster, Lycoming, and Clinton counties, PA in May 2018	Pyrethroid-resistant but unknown mechanisms. Known prior exposure to other pyrethroids, methomyl, imidacloprid, and/or cyromazine	4.49 ± 0.10
	KS8S3	Mixture of FL field populations in 2009	Imidacloprid-resistant due to overexpression of glutathione *S*-transferase and a galactosyltransferase-like gene^58,59^	1.56 ± 0.05

## Results

### Horn flies and stable flies: topical toxicity of fluralaner and permethrin

There was a dose-dependent response to fluralaner at 24 h in both the horn flies and the stable flies (Table 2) and that response increased at 48 h, whereas response to permethrin was largely unchanged. For the horn flies at 24 h, fluralaner was 2.3 times as toxic as permethrin. The toxicity of fluralaner increased 1.6 times at 48 h compared to its 24 h value. For the stable flies at 24 h, permethrin was 45.4 times as toxic compared to fluralaner. Based on the two time points it was measured at, fluralaner was 2.5 times as toxic at 48 h compared to at 24 h**.**

**Table 2:** Topical toxicity of fluralaner and permethrin on horn flies and stable flies at 24 and 48 h.

Species		Insecticide	n	Slope (SE)	LD_50_ (95% CI) (ng/fly)	χ^2^ (p-value)
Horn flies	24 h	Permethrin	400	3.5 (0.3)	10.4 (9.44–11.6)	0.5 (0.8)
	24 h	Fluralaner	320	4.0 (0.4)	4.62 (4.16–5.14)	1.4 (0.5)
	48 h	Fluralaner	320	3.1 (0.4)	2.86 (2.46–3.32)	2.0 (0.2)
Stable flies	24 h	Permethrin	440	3.5 (0.3)	0.76 (0.68–0.83)	2.0 (0.4)
	24 h	Fluralaner	520	2.0 (0.2)	34.5 (29.6–39.9)	5.6 (0.1)
	48 h	Fluralaner	520	2.1 (0.2)	13.9 (11.7–15.9)	1.6 (0.7)

Non-overlap of 95% CI is interpreted as a statistically significant difference.

### House flies: NIU strain sequencing for kdr mutation

The NIU strain, which was the susceptible strain used in the present study, was homozygous susceptible with no L1014F/H mutation detected in any individuals. Only voltage-sensitive sodium channel (*Vssc)* haplotype v26 was detected. While there are multiple *Vssc* alleles known that can cause resistance in house flies^35^, all of them have a mutation at the L1014 site. Thus, the NIU strain does not contain any *kdr* mutation.

### House flies: topical toxicity of fluralaner and permethrin

At 24 h, there was inconsistent and often low mortality in several of the strains, regardless of the fluralaner doses tested (Table 3), but there was a dose-dependent response at 48 h. Thus, 48 h mortality was used to compare LD_50_ values for fluralaner versus permethrin (Table 4). The KS17, USDA-R, USDA-mixed, and PA-mixed strains, were permethrin-resistant relative to the susceptible NIU strain (Table 4). The strain that has been described as imidacloprid-resistant, KS8S3 was 2.6-fold resistant to permethrin (Table 4).

**Table 3:** Mortality at 24 h and 48 h of highest topical dose of fluralaner applied on six strains of house flies*.*

Strain	Fluralaner dose (ng/fly)	24 h mortality (%)	48 h mortality (%)
NIU	35	22	93
KS17	500	27	72
USDA-R	500	78	97
USDA-mixed	1,000	88	98
PA-mixed	400	19	94
KS8S3	100	45	85

**Table 4:** Topical toxicity of fluralaner and permethrin on six strains of house flies at 48 h.

Strain	Insecticide	n	LD_50_ (95% CI) (ng/fly)	Slope (SE)	χ^2^ (p-value)	RR
NIU	Permethrin	240	17.0 (16.2–17.9)	8.4 (1.0)	0.015 (0.90)	–
	Fluralaner	300	17.6 (15.8–19.2)	4.7 (0.6)	0.72 (0.40)	–
KS17	Permethrin	320	> 5,000	–	–	> 290*
	Fluralaner	300	180 (139–243)	1.4 (0.2)	0.27 (0.97)	10*
USDA-R	Permethrin	400	420 (378–467)	3.3 (0.3)	2.8 (0.25)	25*
	Fluralaner	500	46.3 (40.7–52.3)	2.5 (0.2)	2.4 (0.50)	2.6*
USDA-mixed	Permethrin	600	3,080 (2,650–3,570)	1.8 (0.1)	1.68 (0.79)	180*
	Fluralaner	600	115 (101–131)	2.3 (0.2)	0.21 (0.995)	6.5*
PA-mixed	Permethrin	320	719 (540–960)	1.2 (0.2)	5.0 (0.08)	42*
	Fluralaner	320	127 (112–144)	3.1 (0.3)	0.17 (0.92)	7.2*
KS8S3	Permethrin	320	44.3 (36.6–52.5)	2.0 (0.2)	1.9 (0.39)	2.6*
	Fluralaner	320	52.2 (45.9–60.1)	3.0 (0.3)	4.4 (0.11)	3.0*

*RR* Resistance ratio = LD_50_ of resistant or field strain divided by LD_50_ of NIU susceptible strain.

*No overlap of the 95% CI of the LD_50_ with that of the susceptible NIU strain for the same pesticide; i.e., level of resistance-susceptibility differs from NIU. For the RR > 2,900, * is assumed given the extreme magnitude of the difference in LD_50_ and the variance seen for the other LD_50_ values.

Fluralaner was as toxic as permethrin to the NIU susceptible strain and the KS8S3 strain, respectively, but was more toxic than permethrin to the other strains. Specifically, fluralaner was more than 27–28 times as toxic as permethrin to the USDA-mixed strain and KS17 strain, nine times as toxic to the USDA-R strain, and six times as toxic to the PA-mixed strain (Table 4).

Some cross-resistance between topically applied fluralaner and permethrin was observed in all the insecticide-resistant strains tested, as evidenced by all these strains being significantly more resistant than the susceptible NIU strain for both fluralaner and permethrin (Table 4). However, the levels of resistance to fluralaner were much lower than the levels of permethrin resistance in four of the five insecticide-resistant strains (KS17, USDA-R, PA-mixed, and USDA-mixed, but not KS8S3).

The order of susceptibility for topical fluralaner was NIU > USDA-R = KS8S3 > USDA-mixed = PA-mixed = KS17 (USDA-mixed > KS17). For topical permethrin, the order was NIU > KS8S3 > USDA-R > PA-mixed > USDA-mixed > KS17.

### House flies: oral toxicity of fluralaner and imidacloprid

Although only one of the three resistant strains that we tested with imidacloprid, KS8S3, has been described as imidacloprid-resistant (Table 1), both it and USDA-mixed were imidacloprid-resistant relative to the susceptible NIU strain, although not very strongly as indicated by the significant but small RR values of 3.4- and 1.5-fold, respectively (Table 5). USDA-R was not imidacloprid-resistant.

**Table 5:** Oral toxicity of fluralaner and imidacloprid in four house fly strains at 24 h.

Strain	Insecticide	n	LC_50_ (95% CI) (μg/g sugar)	Slope (SE)	χ^2^ (p-value)	RR
NIU	Imidacloprid	320	51.4 (43.6–60.4)	2.2 (0.3)	1.75 (0.42)	–
	Fluralaner	320	2.25 (1.90–2.60)	2.6 (0.3)	4.04 (0.13)	–
USDA-R	Imidacloprid	479	62.0 (53.3–71.0)	2.3 (0.2)	2.39 (0.50)	1.2
	Fluralaner	367	6.93 (5.71–8.15)	2.7 (0.3)	0.32 (0.96)	3.1*
USDA-mixed	Imidacloprid	720	75.9 (69.1–83.1)	2.8 (0.2)	3.59 (0.17)	1.5*
	Fluralaner	720	1.68 (1.52–1.84)	2.7 (0.2)	4.25 (0.12)	0.8*
KS8S3	Imidacloprid	320	173 (150–197)	2.8 (0.3)	0.22 (0.90)	3.4*
	Fluralaner	320	1.47 (1.27–1.68)	3.0 (0.3)	0.36 (0.83)	0.7*

*RR* resistance ratio = LC_50_ of resistant or field strain divided by LC_50_ of NIU susceptible strain.

*No overlap of the 95% CI of the LC_50_ with that of the susceptible NIU strain for the same pesticide; i.e., level of resistance-susceptibility differs from NIU.

Fluralaner was much more toxic than imidacloprid to all four of the strains tested (Table 5). It was 23 times as toxic as imidacloprid to the susceptible NIU strain. Fluralaner was also more toxic than imidacloprid to the other strains: 118 times as toxic to the KS8S3 strain, 45 times as toxic to the USDA-mixed strain, and nine times as toxic to the USDA-R strain.

No oral cross-resistance between imidacloprid and fluralaner was seen. For KS8S3 and for USDA-mixed, this is suggested by these strains being, relative to the NIU strain, more resistant to imidacloprid, yet slightly more susceptible to fluralaner (Table 5). For USDA-R this is suggested by the strain, relative to the NIU strain, being resistant to fluralaner but not imidacloprid.

The order of strain susceptibility from most to least for oral fluralaner, was KS8S3 = USDA-mixed > NIU > USDA-R. For oral imidacloprid the order was NIU = USDA-R = USDA-mixed > KS8S3 (NIU > USDA-mixed).

## Discussion

Our results suggest that fluralaner may be effective for new filth fly control products because of its performance against horn flies and multiple strains of house flies, and because its mode of action differs from any filth fly insecticides currently on the market. Horn flies are often considered the most economically damaging of the filth flies^36,37^. Fluralaner was over twice as toxic as permethrin to horn flies at 24 h. Fluralaner being twice as toxic as permethrin against horn flies under laboratory conditions is promising, but usefulness in the field will depend on formulation, persistence in the environment or on-animal, and cost of formulation. Results here suggest that these issues are worth exploring for fluralaner.

In contrast to fluralaner’s greater toxicity to horn flies, fluralaner was about 46 times less toxic to stable flies than permethrin. On the other hand, this amount is still not relatively large in that only about twice as much fluralaner was needed to kill stable flies as house flies, based on comparing susceptible strains with topical application. Oral tests with fluralaner against resistant strains of stable flies would be useful because stable flies feed on nectar in nature^38^ and will eat sugar-based fly bait^39^.

Future work also should address any cross-resistance concerns in horn flies and stable flies. If cross-resistance is absent or low, as it generally was for house flies, then fluralaner will be especially useful against populations that are resistant to other insecticides. Horn flies can rapidly develop resistance to pyrethroids and organophosphates^40,41^. Resistance to permethrin also can rapidly develop under regular usage regimes in stable flies^42^.

Fluralaner was effective as a topical application against house flies. Specifically, fluralaner was more toxic than permethrin to the four strains with the greatest permethrin resistance, and not significantly less toxic for the other two strains, the susceptible NIU strain and the KS8S3 strain, which showed only mild permethrin resistance. However, topical fluralaner appeared to be slow acting on some house fly strains compared to permethrin. This was most apparent from the lack of symptoms of intoxication during the initial 24 h observations. Mortality also was inconsistent at this timepoint, regardless of the range of doses used. In contrast, symptoms of permethrin intoxication usually appeared within minutes of application and mortality was generally consistent at 24 h. This slow acting nature of topical fluralaner for some strains could be an effect of cuticular penetration, pharmacokinetics inside the insect, or some combination. Other slow acting insecticides that have been adopted for filth fly control include cyantraniliprole and spinosad in granular baits, although they are not as widely used as faster acting insecticides, like neonicotinoids and carbamates^43,44^.

Topical cross-resistance between fluralaner and permethrin was seen in all of the insecticide-resistant strains that we tested. However, fluralaner resistance in these strains was at least an order of magnitude lower compared to permethrin resistance in all but one strain. The exception was the KS8S3 strain, where they were nearly identical but low. Among the pyrethroid-resistant strains that were tested, only the KS17 strain’s resistance mechanisms are known. KS17 possesses *kdr* mutations, as well as ace-2 mutations that confer carbamate and organophosphate resistance^19^. KS17 also has mutations in the cytochrome P450 CYP6D1 gene. Given that the mode of action of fluralaner is different from pyrethroids and carbamates/organophosphates, this suggests that cytochrome P450s or other detoxification enzymes may play some role in cross-resistance of fluralaner in some pyrethroid-resistant house fly strains.

Orally administered fluralaner also appears to be a strong alternative insecticide against house flies with diverse resistance backgrounds. In all four house fly strains that we tested, fluralaner was more toxic than imidacloprid, such that only 0.8–11% as much fluralaner as imidacloprid was needed for 50% mortality at 24 h. Also encouraging was the lack of cross-resistance between imidacloprid and fluralaner. Relative to the susceptible NIU strain, KS8S3 and USDA-mixed strains were resistant to imidacloprid but susceptible to fluralaner. Thus, the mechanisms of resistance to imidacloprid in these two strains do not confer cross-resistance to oral fluralaner in house flies. The mechanism of resistance is unknown for the USDA-mixed strain but imidacloprid resistance in KS8S3 is due to overexpression of glutathione *S*-transferase and galactosyltransferase-like genes.

Sprays, paint-ons, pourables, and baits are not the only ways that fluralaner might be formulated. Ivermectin, like fluralaner, is a chloride channel allosteric modulator that appears to be relatively safe for mammals. Ivermectin has been delivered as livestock injections, boluses, and medicated feed^45–47^. For horn flies and stable flies, such ivermectin applications appear to cause some adult mortality through blood meals, as well as some mortality of the larval stage as a result of livestock defecation^48,49^. The efficacy of fluralaner against horn flies and stable flies in these types of applications remains to be tested.

Our results suggest fluralaner has tremendous potential as a space spray or on-animal treatment for horn flies. These findings could provide a significant advancement in chemical control of horn flies because only a very limited number of insecticides are labeled for their control. Our topical data also suggest that fluralaner has potential as a component of sprays for house flies, particularly for resistant populations. Our oral data suggest that fluralaner has potential as a bait component for both resistant and susceptible populations of house flies. Future work to develop fluralaner-based filth fly control products will involve addressing aspects of EPA registration such as human and non-target toxicology, environmental fate, and physical chemistry. To date, fluralaner appears to pose minimal safety risks for mammals, including humans^34^. The FDA has approved fluralaner as an orally administered systemic for control of blood-feeding arthropods in livestock. The environmental fate of fluralaner has yet to be determined and will be crucial information for future formulation efforts.

## Materials and methods

### Insects and chemicals

Strain information for tested filth flies is in Table 1.

The fluralaner (99.5%) was from BOC Science (Shirley, NY, USA), the imidacloprid (99.5% purity) was from Chem Service (West Chester, PA, USA). The permethrin (99.5%; 19.1% *cis*, 80.9% *trans* isomer ratio) was from Chem Service (West Chester, PA, USA) except for that used with the USDA-mixed house flies, stable flies, and horn flies. Permethrin for the latter was technical grade kept in stock at USDA-CMAVE, but of unknown source, NMR was used to confirm that this permethrin was > 95%; 44.4% *cis*, 55.6% *trans* isomer ratio. Pesticide-grade acetone and methanol were from Fisher Scientific (Waltham, MA, USA).

### Horn flies: topical toxicity of fluralaner and permethrin

Flies used in this study were obtained from a colony of insecticide-susceptible horn flies reared at the USDA-ARS Knipling-Bushland US Livestock Insects Research Laboratory, Kerrville, TX. This colony of horn flies was started with horn flies collected from Lake Jackson, TX in 1968, with no subsequent introductions of outside flies. Colony horn fly larvae were reared on a cow manure and peanut hull diet^50^. After adult emergence, adult horn flies used for the bioassays were held at 24–28 °C, 60% RH, and a photoperiod of 12:12 (L:D) h for 3 days. During this time, the adult flies were fed citrated bovine blood obtained from a local abattoir.

At 3 days post-emergence, adult flies were collected into a glass conical flask from the rearing cage using a vacuum pump. The flies were then knocked down with CO_2_, and the flask was stoppered to allow for transport and transfer to a cold table. Knocked down flies were then transferred to a cold table and were kept immobile by holding them at 4 °C while the flies were sorted into males and females. Sorted females were then counted into groups of 20 flies per treatment dose. Female flies were then individually treated with 0.5 µl of each treatment dose applied topically to the center of the thorax using a Burkard Hand-Operated micro-applicator (Burkard Scientific Ltd., Uxbridge, UK) with a 1 ml glass syringe and a G25 × 25 mm stainless steel needle (Burkard Scientific Ltd., Uxbridge, UK). During treatment, flies were gently held by the wing with fine point micro dissecting forceps, and flies were held briefly for the treatment to dry before the treated fly was placed in a cage. Treated and control flies were held in cages at room temperature and humidity and a photoperiod of 12:12 (L:D) h. Control and treated flies were fed twice daily using citrated bovine blood and cotton sanitary napkin pads cut into 3 cm × 3 cm squares.

### Stable flies: topical toxicity of fluralaner and permethrin

Flies that had emerged < 24 h previously and had never fed were aspirated from colony cages and anesthetized with CO_2_. Flies were separated into groups of 20 females using a magnified illuminator, then transferred to a 14 cm dia. disk of filter paper and treated individually with one 0.5 µl droplet of test solution applied to the pronotum. Applications were with a Hamilton 25 µl gastight glass syringe with a cemented needle and a Hamilton PB-600 Repeating Dispenser to deliver 0.5 µl droplets. Separate syringes were used for acetone-only controls, permethrin, and fluralaner solutions in acetone. Treated flies were transferred to 163 ml plastic cups covered with polyester organza mesh tops. A cotton ball soaked with 10% sucrose was placed on the mesh top of each cup. Cups were placed in environmentally controlled chamber (Percival I36-VL) and held at 25 °C under constant light. Dead flies were counted at 24 h post-treatment for permethrin and at 24 and 48 h post-treatment for fluralaner groups. Flies were scored as “dead” when they were either motionless or unable to move in a controlled manner (i.e., twitching or able only to stumble for a step or two). Syringes were flushed ten times with acetone after delivering test solutions before being used for subsequent treatments.

### House flies: NIU strain sequencing for kdr mutation

The NIU house fly strain has been assumed to be susceptible based on a previous toxicity study and because it has been maintained for > 20 yr. with no exposure to pesticides^51^. Here, we tested the strain for *kdr* mutation. DNA was extracted from single hind legs of eight individual females and seven individual males, using an alkaline extraction^19^. Extracted DNA was stored at − 20 °C. PCR was carried out using 12.5 μL of GoTaq 2x (Promega, Madison, WI, USA), 9.5 μL nuclease free water, 1 μL template DNA, and 1 μL each of the 10 µM forward kdrFL and reverse MdSCR7 primers^19^. Thermal cycler [Bio-Rad T100 (Bio-Rad, Hercules, CA)] conditions were as follows: 95 °C for 3 min, followed by 35 cycles of PCR (95 °C for 30 s, 55 °C for 20 s and 72 °C for 20 s) and a final extension of 5 min at 72 °C. Sanger sequencing of the PCR products was performed by Cornell’s Biotechnology Resource Center, using the primers mentioned above. Electropherograms were manually inspected for resistance mutations.

### House flies: topical toxicity of fluralaner and permethrin

Topical LD_50_ values were determined according to Scott^52^, but with some modification. Between three and five test solutions were created for each AI, each to a volume of 500 μL, in a range that produced > 0% mortality at the lowest dose and < 100% mortality at the highest dose. Twenty 0–1 day old, water-fed female flies were used. They were kept in a Pyrex petri dish (9 cm dia. × 1.5 cm height) in ice until treatment. Each fly was treated with a 0.5 μL droplet of pesticide solution or control, by application to the dorsal side of the thoracic notum (scutum) using a Hamilton 25 μL gastight glass syringe in a Hamilton PB-600 Repeating Dispenser (Hamilton Company, Reno, NV, USA). The flies were then placed in a clean 300 mL glass jar and secured with mesh screen. A piece of cotton soaked in water was placed on top of the screen as a water source, and 200 mg of granular sucrose was poured into to the bottom of the jar. Flies were held at 28 °C, and 12:12 h light/dark. Assessment of mortality initially was at 24 h, but neither symptoms of intoxication nor mortality appeared consistently at this timepoint, regardless of dose range used. Instead, 48 h was the earliest observed timepoint where these measurements were consistent and dose-dependent, thus doses were optimized for 48 h mortality. Each dose and control were replicated at least four times, and data from all replicates were pooled for analysis.

### House flies: oral toxicity of fluralaner and imidacloprid

Determination of oral LC_50_ values followed methods in Burgess and King^51^, but at 24 h instead of 48 h. Briefly, test solutions were created from a stock solution of active ingredient (AI) in acetone using a combination of serial and parallel dilutions. The test solutions chosen produced > 0% mortality at the lowest concentration and < 100% mortality at the highest concentration. Each test solution was made to a total volume of 1,000 μL. For each AI, four or five test solutions were made plus a control of acetone only. A 3.5 g sugar cube (Domino Foods, Inc., Yonkers, NY) was placed inside a 300 mL glass jar, and 500 μL of one of the test solutions or control was pipetted onto the sugar cube. The treated sugar cubes were left for at least 1 h to allow the acetone to completely evaporate. Twenty female flies aged 0–1 days that had been maintained on water-only prior to experimentation were anesthetized with CO_2_ and added to each jar. Flies were held in an environmental chamber at 28 °C and 12:12 h light/dark for 24 h, at which point mortality was assessed. Mortality was defined as being non-responsive when the glass jar was vigorously shaken. Data from all replicates were pooled for statistical analysis. Each set of five test solutions and control were replicated at least three times for each AI.

### Statistical analyses

In computing LD_50_ and LC_50_ values, Abbott’s formula was used to correct for control mortality when applicable^53^. All LD_50_ and LC_50_ values were corrected for AI purity. All analyses were with SPSS^54^.
